# Rehabilitation Strategies for Wrist Pain in a Patient With Thalassemia Major and Distal Ulnar Hypoplasia: A Case Report

**DOI:** 10.7759/cureus.55689

**Published:** 2024-03-06

**Authors:** Grisha Ratnani, Pratik Phansopkar, Harsh R Nathani

**Affiliations:** 1 Musculoskeletal Physiotherapy, Ravi Nair Physiotherapy College, Datta Meghe Institute of Higher Education and Research, Wardha, IND

**Keywords:** physiotherapy, wrist rehabilitation, wrist pain, distal ulnar hypoplasia, thalassemia

## Abstract

This case study examines the rehabilitation process of a 24-year-old female patient with thalassemia major (TM), a hereditary hemoglobinopathy, who also suffered from distal ulnar hypoplasia, a congenital anomaly that causes pain and affects the wrist joint's strength and range of motion. The patient underwent a comprehensive physical rehabilitation program that aimed to address the challenges posed by ulnar hypoplasia. This program included a combination of customized exercises, splinting, and orthotic interventions to improve hand and wrist function. By adopting a multidisciplinary approach, the patient experienced significant improvements in mobility, strength, and overall quality of life. This case highlights the significance of personalized rehabilitation strategies in managing complex medical conditions, demonstrating the potential for positive outcomes even in patients with dual diagnoses of TM and ulnar hypoplasia.

## Introduction

Beta-thalassemia major (β-TM), also referred to as Cooley's anemia or Mediterranean anemia, was initially recorded in 1925 by Cooley and Lee. This particular condition is highly prevalent among populations residing in the Mediterranean basin. Beta-thalassemia is a hereditary hematologic condition that is distinguished by scarcity or total lack of the production of beta-hemoglobin chains, resulting in ineffective erythropoiesis. If left untreated, individuals with βTM experience severe anemia, hepatosplenomegaly, various bone deformities, and stunted growth and typically succumb to heart failure within the first 10 years of life [1]. In patients with untreated or poorly transfused βTM, skeletal changes primarily occur due to excessive production of red blood cells, known as erythroid hyperplasia, which is a result of ineffective erythropoiesis. This abnormal proliferation of bone marrow affects both the outer and inner layers of bones, leading to an expansion of the medullary space. In untreated patients, the bone marrow can expand by a factor of up to 15 to 30. Additionally, there is a thinning of the outer layer of bones and resorption of the secondary and tertiary bone trabeculae, which are replaced by prominent and coarse primary trabeculae. This gives the bones a *lace-like* appearance. Overall, there is a generalized decrease in bone density, leading to osteopenia or osteoporosis [2-5].

Before the introduction of chelation therapy, the absence of iron chelation during repeated transfusions led to the development of hemosiderosis, where iron was deposited in different locations, resulting in both structural and functional impairments. To mitigate the risk of visceral and cardiac toxicity, it is crucial to administer iron chelation therapy. Currently, there are several available agents for iron chelation, such as deferoxamine, deferasirox, and deferiprone. Patients with thalassemia commonly experience arthropathies and bone deformities, which have been attributed to either the disease itself or its treatment [6,7].

Ulnar deficiency, a rare congenital anomaly of the upper extremity, falls under the subcategory of longitudinal ray deficiency in the classification of congenital limb malformations. Among all longitudinal ray deficiencies, the clinical manifestations of ulnar ray deficiency exhibit a broader range of variations [8]. A wide range of musculoskeletal abnormalities in the upper and lower limbs are linked to congenital ulna deficiency. To manage this condition effectively, it is crucial to conduct a thorough physical examination along with the use of radiographs. Various classifications have been developed, focusing on deficiencies in the elbow, forearm, carpal bones, fingers, thumb, and the first web space. Type I represents a mild form of deficiency characterized by the presence of a normal first webspace and thumb, while ulnar digits and carpus are absent [9]. Negative ulnar variance is a frequently seen condition where the ulna bone is shorter than the radius bone. It is often present from birth and is found in about 23% of healthy individuals. However, it can also occur as a result of early closure of the growth plate, injury, or surgery. Even a small negative ulnar variance of 2.5 mm can have a significant impact, reducing the load on the ulna bone from 18% to 4% and shifting most of the force onto the radius bone [10].

## Case presentation

Patient information

**Figure 1:**
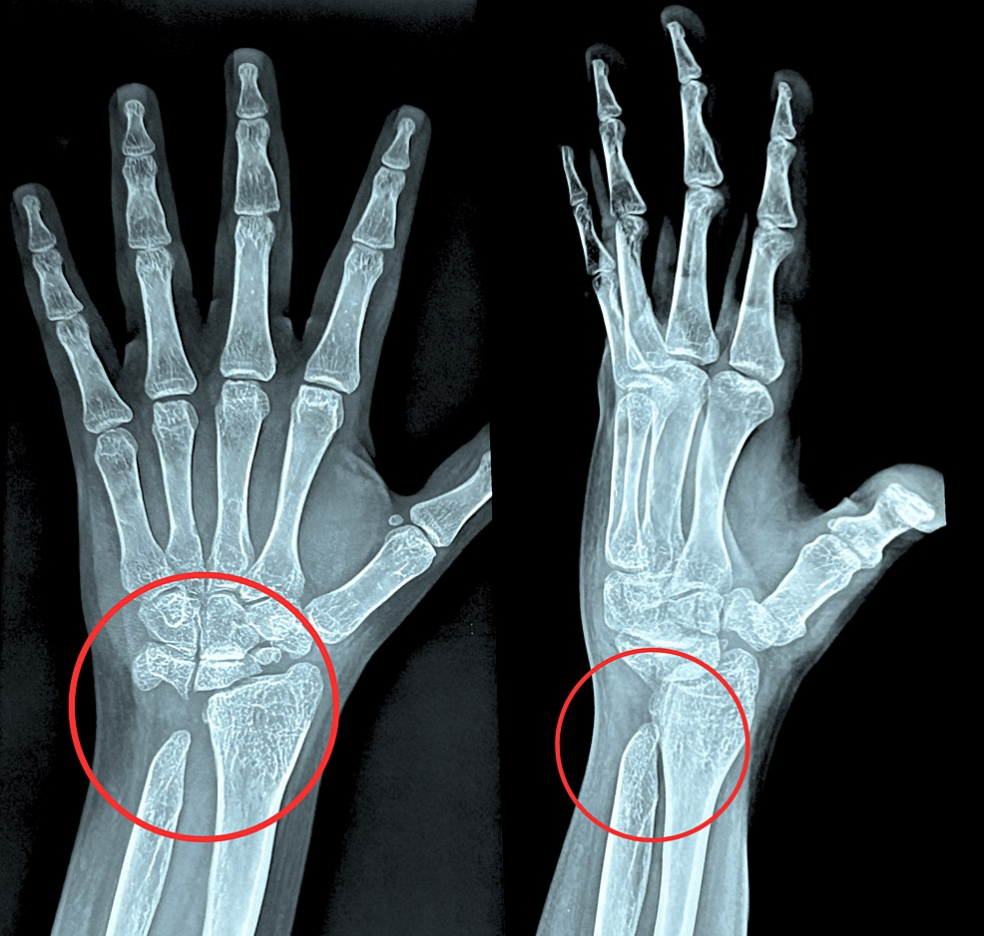
Posteroanterior and lateral views of left wrist radiographic images. The red circle shows an absence of the left ulnar styloid process, indicating distal ulnar hypoplasia as well as fragmented scaphoid.

This report presents a case of a 24-year-old female, 150 cm tall, weighing about 52 kg, right-hand dominant, and an artist by profession who visited the orthopedic hospital with complaints of severe pain in her left wrist along with swelling in the past three days. She reported no history of trauma. The patient is a known case of TM, taking two units of blood transfusion every 21 days for three months of birth. She also mentioned taking iron chelation therapy through medications (Deferasirox 500 mg). She gave no history of trauma, fall, or any injury to the wrist. She was advised of radiographic investigations, which revealed distal ulna hypoplasia and signs of osteoporosis. She was prescribed analgesic medicines along with calcium and multivitamin tablets and was referred for physiotherapy for further management. Figure 1 shows the diagnostic investigation.

Clinical evaluation

During the clinical examination, the patient expressed a pain level of 8 out of 10 on the numerical pain rating scale (NPRS). Upon assessment, it was observed that the strength and mobility of the affected upper limb were diminished when compared to the normative ranges specified in Tables 1-2. The evaluation of grip strength was conducted using a handheld dynamometer. The end feeling was determined to be empty as a result of the pain experienced by the patient.

**Table 1:** Range of motion of affected (left) hand and wrist.

	Passive	Active
Wrist flexion	30°	20°
Wrist extension	35°	15°
Ulnar deviation	10°	10°
Radial deviation	10°	10°
Pronation	40°	25°
Supination	40°	20°
Elbow flexion	130°	135°

**Table 2:** Manual muscle testing of affected musculature according to modified Oxford grading. 3-: (Fair-) Some but not complete range of motion against gravity.

Muscles	Grades
Wrist flexors	3-
Wrist extensor	3-
Pronators	3-
Supinators	3-

Management

Physiotherapy rehabilitation was complemented by orthopedic treatment, which involved the administration of specific medications such as Nusidol serratiopeptidase tablets, Pantoprazole tablets, Tripoheal tablets, and Oscalbon tablets. The physiotherapy sessions were conducted five times a week, with each session lasting approximately 40 minutes for four weeks. Toward the end of the fourth week, the patient was instructed on a home program to continue their rehabilitation. Table 3 consists of the physiotherapeutic intervention administered to the patient.

**Table 3:** Physiotherapy intervention protocol. DAPRE, daily adjustable progressive resistive exercise; Hz, hertz; W/m^2^, watt per square meter

Phases	Goals	Physiotherapy intervention	Dosage
Week 1	Patient education and counseling	The patient was provided with information regarding the significance of physical activity, as well as the necessity for taking precautions and elucidating the benefits of exercise to enhance their condition.	At the beginning of the physiotherapeutic session
	To manage pain and swelling	Cryotherapy was administered at the site of pain.	10-12 minutes thrice a day for one week
		Therapeutic ultrasound	Mode: Pulse (1:4); intensity 0.8 W/m^2^; frequency 3 Hz
	To provide stability to surrounding structures	Wrist forearm brace	The patient was instructed to wear a brace throughout the day for the first week of rehabilitation.
	To regain the range of motion of the wrist and prevent stiffness	Active range-of-motion exercises in available pain-free ranges, including wrist flexion, extension, supination-pronation, ulnar, and radial deviations. Opening and making a fist.	One set of 10 repetitions
Week 2-3	To improve wrist and elbow joint range of motion	Muscle energy technique for wrist flexors, extensors, and elbow flexors	10 repetitions for one set
		Wrist-active range-of-motion exercises	Two sets of 10 repetitions
	To improve the grip strength	Making a fist, compressing a gel ball.	One set of 10 repetitions for the second week, gradually progressing to two sets for the third week
		Opening of fingers against the resistance of web exerciser.	
Week 4	To increase the strength of wrist flexors, extensors, supinators, and pronators	Resistance training using a dumbbell	According to the DAPRE principle of resistance training
	To maintain and increase the muscle length	Stretching of wrist flexors and extensors	Three repetitions withhold for 30 seconds
	To return to work	Functional, task-oriented training	Three sets of 10 repetitions

Educating and counseling patients play crucial roles in providing comprehensive care for individuals with thalassemia who experience skeletal abnormalities. These practices facilitate informed decision-making, enhance emotional well-being, and improve the overall quality of life for both patients and their families. The utilization of cold therapy has been found to effectively diminish inflammatory reactions, leading to a reduction in swelling and ultimately providing relief from pain [11]. The muscle energy technique improves muscle flexibility by enhancing endurance to stretching, resulting in decreased perception of pain (hypoalgesia) through the activation of muscle and joint mechanoreceptors. These mechanoreceptors include the periaqueductal grey as well as the non-opioid serotonergic and noradrenergic descending inhibitory pathways [12]. Motor control experiences a significant improvement within the initial two weeks of resistance training, while enhancement in wrist strength is observed within the first four weeks of resistance training [13]. Ulnar hypoplasia may lead to instability within the wrist joint and forearm, causing potential complications. The utilization of a splint can offer external support, effectively stabilizing the affected region and mitigating the risk of excessive or irregular movements. This approach aids in alleviating discomfort and minimizing the likelihood of additional joint problems.

Follow-up and outcome measures

The patient underwent assessment during the second and third weeks of the intervention. She stated that her pain had diminished to 1/10 by the second week, as per the NPRS. Additionally, there were notable improvements in both range of motion and strength. Detailed results can be found in Tables 4-5.

**Table 4:** Assessment of range of motion after the second and fourth weeks.

Joint	Range of motion		
	Preintervention	Second-week follow-up	Fourth-week follow-up
Wrist joint			
Flexion	0°-30°	0°-55°	0°-75°
Extension	0°-35°	0°-60°	0°-70°
Ulnar deviation	0°-10°	0°-20°	0°-30°
Radial deviation	0°-10°	0°-15°	0°-20°
Elbow joint			
Flexion	0°-130°	0°-140°	0°-140°
Forearm			
Pronation	0°-40°	0°-55°	0°-75°
Supination	0°-40°	0°-60°	0°-75°

**Table 5:** Evaluation of manual muscle testing after the second and fourth weeks of intervention. 3- (Fair-): Some but not complete ROM against gravity 3+ (Fair+): Complete ROM against gravity with minimal resistance 5+ (Normal): (100%) Complete ROM against gravity with maximal resistance 4 (Good): (75%) Complete ROM against gravity with some (moderate) resistance ROM, range of motion

Muscles	Muscle strength according to modified Oxford grading		
	Pre-intervention	Second-week follow-up	Fourth-week follow-up
Wrist flexors	3-	3+	4
Wrist extensor	3-	3+	4
Pronators	3-	4	5+
Supinators	3-	4	5+

## Discussion

The presented case report describes a 24-year-old female patient with thalassemia and ulnar hypoplasia, a congenital condition characterized by the underdevelopment of the ulna bone in the medial aspect of the forearm. The key findings of the case included discomfort around the wrist, limited range of motion, and associated functional impairments. The current approach to treating TM involves the administration of blood transfusions to maintain adequate levels of hemoglobin. Additionally, chelation therapy is employed to eliminate excess iron stores resulting from frequent transfusions.

A study observed growth disturbance and radiologic changes in the long bone metaphyses of patients with TM who underwent both hyper-transfusions and chelation therapy. The researchers suggested that these abnormalities may be attributed to the early initiation of chelation therapy using deferoxamine. They hypothesized that the drug could have a direct toxic effect on bone growth or that the loss of minerals other than iron, or a combination of both factors, could be responsible for these effects [14]. Despite regular transfusions, adequate hormone replacement, and effective iron chelation therapy resulting in the normalization of hemoglobin levels, individuals diagnosed with β-TM still encounter an imbalanced bone turnover. This imbalance is marked by an increased resorptive phase, leading to a significant reduction in bone mass density (BMD) and the development of osteoporosis. Consequently, the risk of fractures, deformities, and chronic bone pain is heightened. Dysplastic alterations frequently affect the spine and long bones, potentially causing growth retardation. Notably, patients who have undergone iron chelation therapy for more than three years are more prone to experiencing dysplastic characteristics, both in terms of frequency and severity. Approximately 50% of individuals with transfusion-dependent thalassemia eventually develop osteoporosis, with their BMD continuously and significantly declining over time [15].

The study conducted by Dhawan et al. aimed to investigate the skeletal changes in the wrist joints of children with transfusion-dependent thalassemia. The study focused on examining the correlations between these changes and various factors such as age, pretransfusion hemoglobin levels, serum ferritin levels, and the types and durations of chelation therapy. Clinical examinations and radiologic assessments were performed to assess the skeletal changes in the wrist joints. The results of the study revealed significant discrepancies in the length of the radial and ulnar bones with age, with a noticeable shortening of the ulnar bone. Furthermore, a correlation was observed between negative ulnar variance and the distal radial articular angle, particularly in cases where deferiprone was used as a chelation therapy. This finding suggests a potential association between ulnar growth arrest and radial bowing. Additionally, the study evaluated the range of motion in the wrist joints and found that it was decreased compared to the normal group. These findings provide valuable insights into the skeletal changes and potential complications associated with transfusion-dependent thalassemia in children [16]. Physiotherapeutic interventions play a crucial role in enhancing the functionality of the affected hand and mitigating the risk of additional injuries caused by abnormal skeletal structure, particularly when surgical interventions can be avoided. This case report presents compelling evidence of enhanced outcome measures following a wrist injury in a patient diagnosed with distal ulnar hypoplasia.

## Conclusions

This case report highlights the successful implementation of a tailored physical rehabilitation program for a patient with TM and ulnar hypoplasia. The patient experienced significant improvements in mobility, strength, and overall functionality. This emphasizes the importance of addressing the complexities of dual diagnoses and advocating for a patient-centered approach to healthcare. Further research in similar populations could lead to standardized protocols and improved outcomes for individuals with these medical challenges.
